# Glibenclamide-Loaded Nanoparticles Reduce NLRP3 Inflammasome Activation and Modulate miR-223-3p/miR-7-1-5p Expression in THP-1 Cells

**DOI:** 10.3390/ph16111590

**Published:** 2023-11-10

**Authors:** Roberta Mancuso, Lorenzo Agostino Citterio, Simone Agostini, Ivana Marventano, Francesca La Rosa, Francesca Re, Pierfausto Seneci, Marina Saresella, Mario Clerici

**Affiliations:** 1 IRCCS Fondazione Don Gnocchi—ONLUS, 20148 Milan, Italy; rmancuso@dongnocchi.it (R.M.); lcitterio@dongnocchi.it (L.A.C.); imarventano@dongnocchi.it (I.M.); flarosa@dongnocchi.it (F.L.R.); msaresella@dongnocchi.it (M.S.); mario.clerici@unimi.it (M.C.); 2 School of Medicine and Surgery, University of Milano-Bicocca, 20854 Milan, Italy; francesca.re1@unimib.it; 3 Dipartimento di Chimica, University of Milan, 20122 Milan, Italy; pierfausto.seneci@unimi.it; 4 Department of Pathophysiology and Transplantation, University of Milan, 20122 Milan, Italy

**Keywords:** glibenclamide, nanoparticles, NLRP3 inflammasome, microRNAs, rehabilitation

## Abstract

The anti-hyperglycemic drug glibenclamide (Glb) might represent an interesting therapeutic option in human neurodegenerative diseases because of its anti-inflammatory activity and its ability to downregulate activation of the NLRP3 inflammasome. Bi-functionalized liposomes that can cross the blood–brain barrier (BBB) may be used to release Glb into the central nervous system (CNS), overcoming its poor solubility and bioavailability. Here, we analyzed in vitro the effect of Glb-loaded nanovectors (GNVs) and Glb itself on NLRP3 inflammasome activation using a lipopolysaccharide- and nigericine-activated THP-1 cell model. Apoptosis-associated speck-like protein containing a CARD (ASC) aggregation and NLRP3-related cytokine (IL-1β, caspase 1, and IL-18) production and gene expression, as well as the concentration of miR-223-3p and miR-7-1-5p, known to modulate the NLRP3 inflammasome, were evaluated in all conditions. Results showed that both GNVs and Glb reduced significantly ASC-speck oligomerization, transcription and translation of NLRP3, as well as the secretion of caspase 1 and IL-1β (*p* < 0.05 for all). Unexpectedly, GNVs/Glb significantly suppressed miR-223-3p and upregulated miR-7-1-5p expression (*p* < 0.01). These preliminary results thus suggest that GNVs, similarly to Glb, are able to dampen NLRP3 inflammasome activation, inflammatory cytokine release, and modulate miR-223-3p/miR-7-1-5p. Although the mechanisms underlying the complex relation among these elements remain to be further investigated, these results can open new roads to the use of GNVs as a novel strategy to reduce inflammasome activation in disease and rehabilitation.

## 1. Introduction

Glibenclamide (Glb), a sulfonylurea widely used as an anti-hyperglycemic agent for type II diabetes mellitus, is endowed with anti-inflammatory effects in multiple clinical conditions (i.e., respiratory diseases, acute pancreatitis, cystitis, ischemia, sepsis), including central nervous system (CNS) diseases [1,2]. However, Glb is poorly water soluble, has a scarce ability to cross the blood–brain barrier (BBB), and is rapidly removed from the CNS. As a consequence of these peculiarities, Glb does not achieve therapeutic levels in brain and cerebrospinal fluid in animal models [3].

The use of innovative nanoparticles represents an interesting option for drug delivery, and different formulations have been developed. Dual-ligand liposomes, in particular, efficiently cross the BBB and can specifically deliver therapeutic molecules into the CNS, thus being a very promising approach for the treatment of neurological diseases [4,5]. In vitro results showed that liposomes loaded with Glb and functionalized with peptides from the receptor-binding domain of apolipoprotein E (mApoE) and matrix metalloproteinase (MMP)-sensitive lipopeptides cross the BBB and release Glb, downregulating the production of proinflammatory cytokines from activated microglial cells [6].

Glb binding to sulfonylurea receptor (Sur1/2) potently inhibits ATP-sensitive ion channels (K^+^ATP) [7] and reduces K^+^ efflux. This results in cell membrane depolarization and inhibits P2X7 receptor-mediated Ca^2+^ influx. Since Glb acts downstream of the P2X7 receptor and upstream of NLRP3, caspase 1 activation and IL-1β production are downregulated [1]. The end product in animal models is a significant reduction in neuroinflammation and neuroprotection after brain injuries [8,9].

The NLRP3 (NACHT, LRR, and PYD domains-containing protein 3) inflammasome is a molecular platform composed of the NLRP3 sensor, the adaptor apoptosis-associated speck-like protein containing a CARD (ASC), and pro-caspase 1. The NLRP3 inflammasome complex assembly occurs in two steps and leads to gasdermin D-mediated pore formation and the release of the proinflammatory cytokines IL-1β and IL-18 [10].

Notably though, although the anti-inflammatory action of Glb involves an interaction with K^+^ ATP-channels [11], its effect can also be observed also in K^+^ ATP-channels-deficient macrophages [1], suggesting the involvement of other mechanisms.

In silico predictions and in vitro/in vivo validation models indicate that miRNAs, and in particular miR-223-3p [12] and miR-7 [13], play a role in reducing NLRP3 translation and inflammasome activation [14]. The aim of this study was to analyze whether Glb and Glb-loaded liposomes (nanovectors with glibenclamide = GNVs) could modulate the activation of the NLRP3 inflammasome. To this end, we analyzed NLRP3-related genes and protein expression as well as miR-223-3p and miR-7-1-5p concentration using an in vitro THP-1 cell model.

## 2. Results

### 2.1. GNVs and Glb Inhibit NLRP3 Inflammasome Activation in THP-1 DMs

THP-1 derived macrophages (DMs) activated in vitro with lipopolysaccharides (LPS) and nigericine were incubated in the absence/presence of either GNVs at a concentration of 10 µM, selected because of previously published results [6], or free Glb (25 µM, i.e., a 4 time lower concentration than reported to be toxic for THP-1 cells) [15]. GNVs and Glb potential toxicity was excluded in every experiment, as cell viability was always >90%. ASC-speck formation, the hallmark of inflammasome activation, was analyzed intracellularly; the production of caspase 1, IL-1β and IL-18, and inflammasome effector proteins was measured in THP-1 DM supernatants.

**Figure 1:**
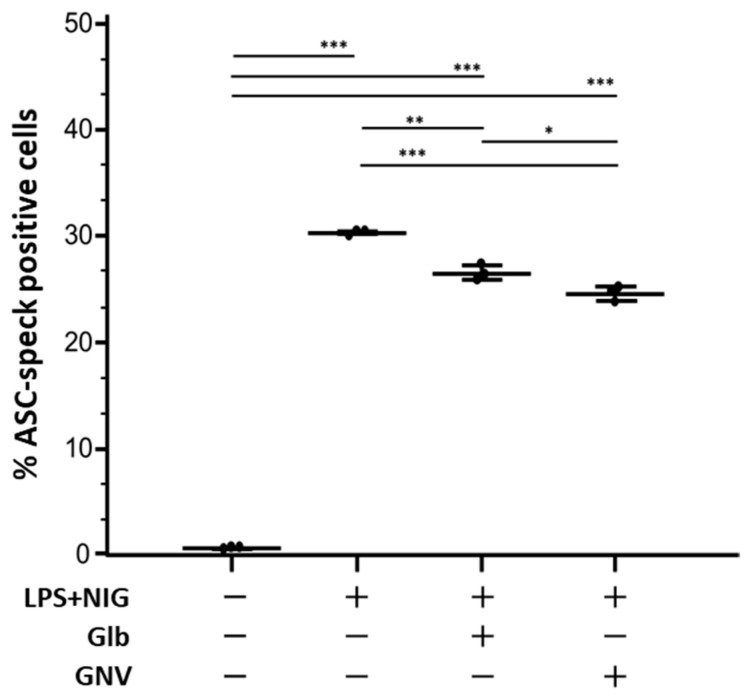
ASC-speck formation. Percentage of THP-1 DMs expressing ASC-speck in unstimulated or LPS+nigericine (NIG)-stimulated cells in presence/absence of glibenclamide (Glb) or glibenclamide-loaded nanovectors (GNVs). Graph represents median values obtained from three independent experiments. Data are expressed as median and interquartile range (25th–75th percentiles). Statistical significance is shown: * = *p* < 0.05; ** = *p* < 0.001; *** *p* < 0.0001.

The percentage of ASC-speck positive cells in THP-1 DMs stimulated in the presence of GNVs (mean: 24.9%) or Glb (26.2%) was significantly reduced compared to that observed in LPS+nigericine-activated THP-1 DMs cultured in medium alone (median: 30.8%; *p* < 0.001 for both comparisons) (Figure 1). Representative dot plot and images of ASC-speck formation are shown in Supplementary Materials, Figure S1.

**Figure 2:**
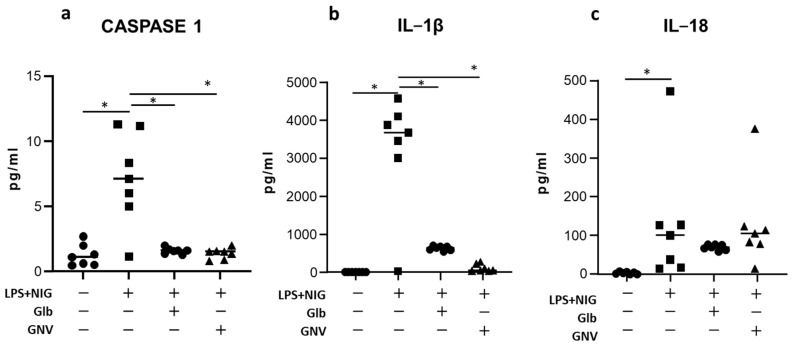
Activated caspase 1, IL-1β, and IL-18 production. (**a**) Activated caspase 1, (**b**) IL-1β, and (**c**) IL-18 production in supernatants of unstimulated or in LPS+nigericine (NIG)-stimulated THP-1 DMs in presence/absence of glibenclamide (Glb) or glibenclamide-loaded nanovectors (GNVs). Data are expressed as pg/mL; graphs represent median value obtained from three independent experiments; dots represent individual values. * = *p* < 0.05.

Caspase 1 and IL-1β concentration in supernatants was significantly reduced as well, when results obtained in THP-1 DMs activated in the presence of GNVs (median: caspase 1: 1.5 pg/mL; IL-1β: 32.0 pg/mL) or Glb (caspase 1: 1.6 pg/mL; IL-1β: 645.0 pg/mL) were compared to those obtained in LPS+nigericine-activated THP-1 DMs (caspase 1: 7.1 pg/mL; IL-1β: 3678.0 pg/mL) (*p* < 0.05 for all comparisons) (Figure 2).

In contrast with these results, the IL-18 concentration was similar in all the experimental conditions analyzed (GNVs, median: 105.0 pg/mL; Glb: 68.6 pg/mL; medium alone: 99.0 pg/mL).

### 2.2. GNVs and Glb Reduce LPS+Nigericine-Induced NLRP3 Gene Expression in THP-1 DMs

To evaluate whether the GNV- and Glb-associated downregulation of NLRP3 inflammasome activation is the result of NLRP3 transcriptional downregulation, mRNA expression was measured using a digital droplet PCR (ddPCR) in LPS+nigericine-activated THP-1 DMs in the presence/absence of GNVs or Glb.

At first, we analyzed the NLRP3 expression in LPS+nigericine-activated THP-1 DMs during 24 h, with or without Glb and GNVs, which showed a rapid and significant increase in mRNA expression within one hour of stimulation (*p* = 0.009 vs. unstimulated), that was still significant 3 and 6 h after activation, decreasing to baseline levels after 24 h. Activation in the presence of either GNVs or Glb completely prevented NLRP3 mRNA expression increases at all considered time points (see Supplementary Materials, Figure S2). Both GNVs and Glb, thus, induce a potent transcriptional downregulation of the NLRP3 gene in activated THP-1 DMs.

**Figure 3:**
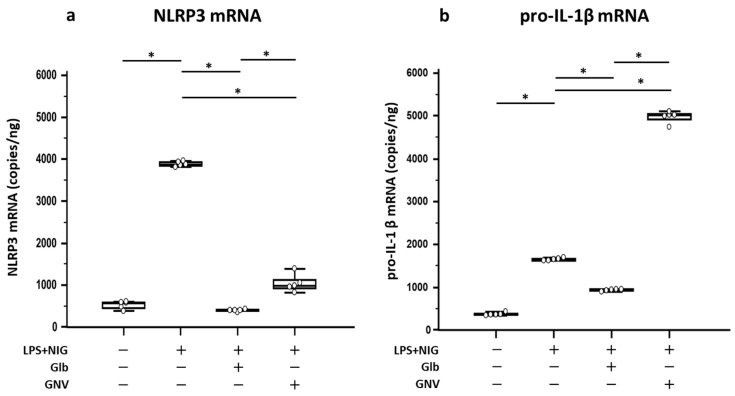
NLRP3 and pro-IL-1β mRNA expression. (**a**) NLRP3 gene expression in THP-1 DMs. NLRP3 mRNA expression in unstimulated or LPS+nigericine (NIG)-stimulated THP-1 DMs in presence/absence of glibenclamide (Glb) or glibenclamide-loaded nanovectors (GNVs). Data are expressed as copies/ng. Graph shows median values and interquartile range (25th–75th percentiles) obtained from three independent experiments. Statistical significance is shown: * = *p* ≤ 0.01. For additional details, see Section 4. (**b**) pro-IL-1β mRNA expression in THP-1 DMs that were cultured in medium alone or LPS+nigericine (NIG) activated in the presence/absence of glibenclamide (Glb) or glibenclamide-loaded nanovectors (GNVs). Data are expressed as copies/ng; graph represents median values and interquartile range (25th–75th percentiles). Statistical significance is shown: * = *p* ≤ 0.01.

In the next series of experiments, NLRP3-specific mRNA expression was evaluated in THP-1 DMs after 1 h of stimulation with LPS+nigericine in the presence/absence of GNVs or Glb. The results shown in Figure 3a confirmed that both agents result in a significant reduction in NLRP3 transcription; remarkably, neither GNVs nor Glb alone had any effect on NLRP3 expression in unstimulated cells. All these data are summarized in Supplementary Materials, Table S1.

### 2.3. GNVs and Glb Reduce NLRP3 Protein Expression in THP-1 DMs

**Figure 4:**
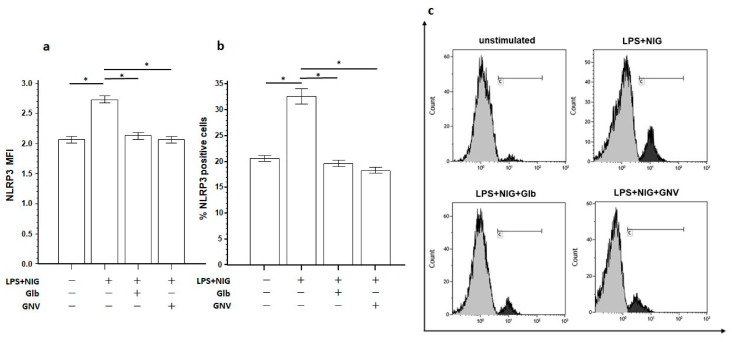
NLRP3 expression. (**a**) Mean fluorescence intensity (MFI) and (**b**) percentage of THP-1 DMs expressing NLRP3 in unstimulated or LPS+nigericine (NIG)-stimulated cells in the presence/absence of glibenclamide (Glb) or glibenclamide-loaded nanovectors (GNVs). Graphs represent mean value and standard deviation obtained from three independent experiments. Statistical significance is shown: * *p* < 0.0001. (**c**) Representative histograms obtained by flow cytometry analysis of THP-1 DMs expressing NLRP3 (%), indicated in gate C, in unstimulated or LPS+NIG-stimulated cells in the presence/absence of Glb or GNVs.

To verify if GNVs or Glb reduce NLRP3 intracellular protein, its expression level was measured by flow cytometry in THP-1 DMs activated with LPS+nigericine for 24 h in the presence/absence of GNVs or Glb. Results showed that both GNVs and Glb significantly reduced the percentage (GNVs = 18.3; Glb = 19.67) and the mean fluorescence intensity (MFI) (GNVs = 2.13; Glb = 2.07) of NLRP3 protein-expressing THP-1 DM cells compared to THP-1 DM-activated cells cultured in medium alone (% = 32.67; MFI = 2.73) (*p* < 0.001 for all the comparisons) (Figure 4).

### 2.4. GNVs and Glb Modulate Pro-IL-1β Expression and IL-1β Intracellular Concentration in THP-1 DMs

Pro-IL-1β mRNA was measured next by ddPCR in THP-1 DMs that had been LPS+nigericine-activated in the presence/absence of GNVs and Glb. Results showed that GNVs and Glb had an opposite effect. In fact, pro-IL-1β mRNA expression was significantly reduced by Glb (*p* = 0.009), but was increased by GNVs, compared to the values observed in activated cells cultured in medium alone (*p* = 0.007) (Figure 3b). These data are summarized in Supplementary Materials, Table S1.

**Figure 5:**
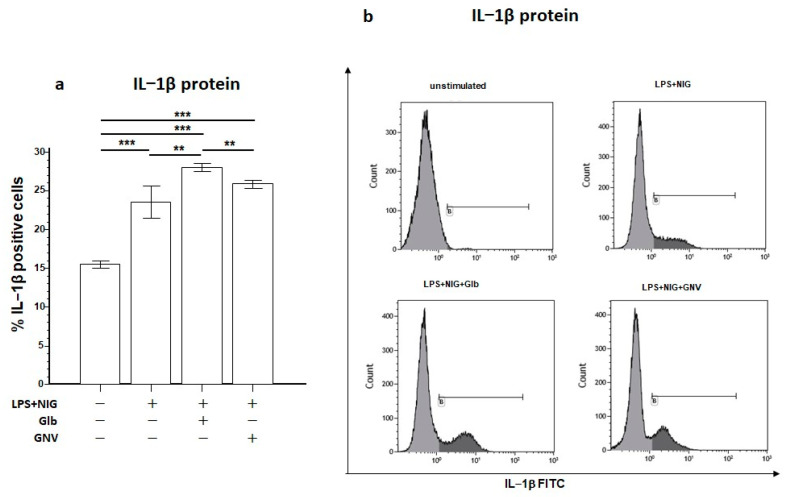
IL-1β. (**a**) Percentage of THP-1 DMs activated in the same culture conditions and expressing IL-1β protein. Graphs represent mean ± SD values obtained from three independent experiments. Statistical significance is shown: *** *p* < 0.001; ** *p* ≤ 0.05. (**b**) Representative histograms obtained by flow cytometry analysis of THP-1 DMs activated in the same culture conditions and expressing IL-1β protein (%), indicated in gate B.

Analysis of the intracellular expression of IL-1β protein showed its increased percentage in LPS+nigericine-activated THP-1 DMs cultured in the presence of either GNVs (mean: 25.9%) or Glb (28.0%), compared to activated cells (23.5%; *p* = 0.02 vs. Glb) (Figure 5a,b). Notably, these results are in stark contrast with the significantly reduced IL-1β concentration observed in THP-1 DMs activated in the presence of GNVs and Glb (see above).

### 2.5. Effect of GNVs and Glb on miR-223-3p and miR-7-1-5p Expression

**Figure 6:**
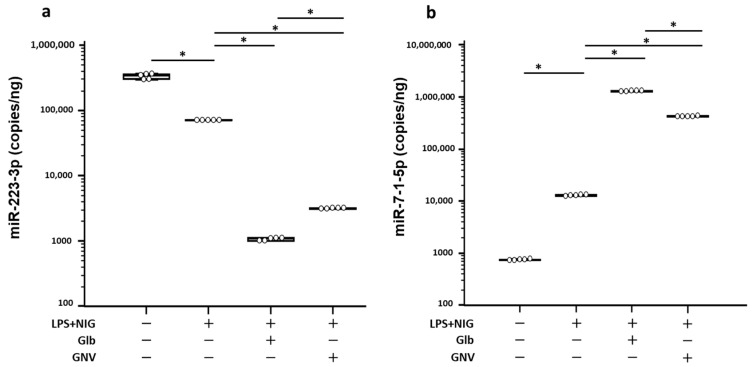
Effect of GNVs on miR-223-3p and miR-7-1-5p expression. Expression (as copies/ng) of miR-223-3p (**a**) and miR-7-1-5p (**b**) measured by ddPCR in THP-1 DMs cultured in medium alone or stimulated with LPS+nigericine (NIG) in presence/absence of glibenclamide (Glb) or glibenclamide-loaded nanovectors (GNVs). Graphs represent median values and interquartile range (25th–75th percentiles) obtained from three independent experiments. Statistical significance is shown: * = *p* 0.01. For additional details, see Section 4.

Finally, we analyzed the ability of GNVs and Glb to modulate the expression of miR-223-3p and miR-7-1-5p in THP-1 DMs that had been LPS+nigericine-activated in the presence/absence of GNVs or Glb (Figure 6); these two mRNAs were chosen because they bind NLRP3-3′UTR and control the activation of the NLRP3 inflammasome [12,13].

Not surprisingly, LPS+nigericine activation resulted in the stimulation of the NLRP3 inflammasome. This was associated with a significant reduction in miR-223-3p concentration (*p* = 0.009), which was further downregulated both by Glb and GNVs. miR-7-1-5p expression behaved in the opposite way, as it was significantly increased in activated cells and was greatly augmented by both GNVs and Glb compared to values observed in LPS+nigericine-activated THP-1 DMs cultured in medium alone (*p* = 0.009 for both compounds). All these data are summarized in Supplementary Materials, Table S2.

## 3. Discussion

GNV liposomes were developed as an attempt to overcome the poor brain bioavailability and systemic side effects of Glb, and were recently shown to have an anti-inflammatory effect as they reduce TNFα and IL-6 production from activated microglia [6]. We verified whether GNVs could downregulate the activation of the NLRP3 inflammasome using the experimental model of LPS+nigericine-activated THP-1 DMs, comparing the results to those obtained using free Glb. Results showed that GNVs significantly reduce ASC oligomerization, the transcription and translation of NLRP3, as well as caspase 1, IL-18, and IL-1β production. Interestingly, GNVs had a discordant effect of the expression of miR-223-3p and miR-7-1-5p, two of the best known miRNAs suggested to modulate NLRP3 gene expression upon binding its 3′ UTR [12,13].

Aberrant activation of the NLRP3 inflammasome in monocytes/microglia has been previously reported in different neurodegenerative diseases [16,17,18]. These data stimulated research on compounds that upon hampering inflammasome activation in microglia would reduce neuroinflammation and neurodegeneration [19,20,21,22].

Glb, currently used for type 2 diabetes treatment, showed an anti-inflammatory activity and a protective role against tissue damage triggered by inflammation not only in the CNS but also in other tissues [2,11]; however, only a limited quantity of Glb reaches the CNS after systemic administration [3]. Glb was shown to bind specifically to the Sur1 receptor, thus inhibiting ATP-sensitive K^+^ channels which are constitutively expressed by different cell types and tissues, including macrophages [23]. In the latter cell type, Glb prevents the cytosolic depletion of potassium, inhibiting Ca^2+^ influx through P2X7 receptors, and suppressing the NLRP3 inflammasome activation [1,24]. It is also known that Glb, by modulating NF-Kb and MAPK signaling [25], acts downstream of the P2X7 receptor and upstream of NLRP3 [1,15,26]. The effect of Glb on NLRP3-related miRNAs is nevertheless mostly unprecedented, as it was only investigated using the expression of NLRP3-related proteins and genes as readouts [1,15,25,26].

GNVs were able to drastically reduce NLRP3 gene and protein expression, as was free Glb, in the latter case confirming previous results [27]. Decreased amounts of NLRP3 protein justify the observed reduction in intracellular ASC-speck complexes observed in our experiments, explaining the hampering effect of these agents on inflammasome activation.

The increased expression of miRNAs (in particular the increased miR-7-1-5p expression) may lead to increased NLRP3 mRNA degradation; otherwise, it is also possible that GNVs/Glb may induce NLRP3 post-translational modifications (in particular, phosphorylation and ubiquitination) [10]. However, the molecular mechanism by which this happens must be further investigated.

The effects of Glb and GNVs on IL-1β are complex. Thus: (1) GNVs, but not free Glb, resulted in a robust increase in pro-IL-1β-specific mRNA; (2) both Glb and GNVs, although to a lesser extent, resulted in an increased percentage of THP-1 DMs containing IL-1β; and (3) both agents significantly reduced IL-1β production. Previous results showed an inhibitory effect of Glb on IL-1β secretion [15], but the different effect of GNVs and Glb on pro-IL-1β transcription is an unexpected finding. IL-1β transcription depends primarily on the Spi1, NF-kB, and C/EBPb transcription factors [28,29], which are highly expressed in activated monocytes. Upon LPS stimulation, inducible pro-IL-1β transcription involves a complex sequence of events including methylation of promoter and chromatin remodeling. We hypothesize that free Glb modulates mRNA degradation more efficiently than the same drug loaded in nanoparticles; alternatively, Glb and GNVs may differently modulate elements directly or indirectly involved in pro-IL-1β transcription. Finally, miRNA may also play a role in this phenomenon; miR-302b, in particular, was shown to modulate IL-1β expression in THP-1 in response to monosodium urate [30]. Further investigations will be needed to better understand this mechanism, considering the detection of pro- and mature IL-1β protein by immunoblotting, and the evaluation of other miRNAs targeting IL-1β.

Glb and GNVs did not downregulate IL-18 secretion by activated THP-1 DMs. It is possible that the relatively low concentration of GNVs used in the experiments may be responsible for such results. Alternatively, although IL-18 secretion depends on caspase 1 activation, we suggest that other factors, including plasma membrane permeability [31] and the activation of non-canonical inflammasomes, could explain IL-18 secretion in the presence of GNVs.

All steps of the complex process of NLRP3 inflammasome activation are highly controlled to avoid hyperactivation and, although these molecular mechanisms are still under investigation, multiple results show that miRNAs play an important role in post-transcriptional control of NLRP3 [32]. To further investigate the possible mechanisms of GNV/Glb-induced NLRP3 inflammasome inhibition, we focused our attention on miR-223-3p and miR-7-1-5p, two miRNAs that target NLRP3. miR-223-3p is a highly conserved miRNA with a key regulatory role in myeloid differentiation and function [33], which is downregulated during monocyte/macrophage differentiation [12]. A reduced concentration of miR-223-3p results in an increased expression of pro-inflammatory cytokines, with a consequent induction of M1 phenotype, whereas its overexpression inhibits LPS-induced macrophage activation [11,12,33].

Consistent with previous data [34], our results show that activation of inflammasome reduces miR-223-3p and increases NLRP3 transcription. GNVs/Glb induced a strong and unexpected suppression of miR-223-3p expression with a concomitant upregulation of miR-7-1-5p expression, resulting in a significant downregulation of NLRP3 transcription. Remarkably, the effect of GNVs on NLRP3 and miRNAs was less significant than Glb. A possible explanation may be the relatively low GNV concentration used in our experiments; other members of the NF-KB pathways may nevertheless be involved in this effect. On the whole, an inverse trend of expression for miR-223-3p and miR-7-1-5p was observed when activated THP-1 DMs were exposed to either GNVs or free Glb, resulting in a decreased translation and secretion of the NLRP3 protein. It is important to consider that many other miRNAs have been suggested to be involved in the control of inflammasome activation [15,33]; analyses of these molecules will be important to attain a more comprehensive picture of this mechanism.

miR-7-1-5p is a highly conserved miRNA [35,36,37] that, amongst its multiple effects, targets NLRP3 expression inducing inflammasome activation [13]. Both free Glb and GNVs upregulated miR-7-1-5p expression; this is consistent with previous data in LPS-stimulated BMDM, showing that miR-7-1-5p affects the TLR4 signaling pathway and the transduction of inflammatory cytokines [38]. miR-7-1-5p also targets the SKT and AMPK kinases, reducing PPARγ (proliferator-activated receptor γ) [39], a nuclear transcription factor that modulates adipose tissue inflammatory responses and systemic insulin sensitivity [40,41]. Interestingly, PPARγ is one of the transcription factors that modulate miR-223-3p expression and an alternate mechanism driving macrophage polarization [42]. The PPARγ/miR-223-3p/miR-7-1-5p axis in THP-1 cells and the effect of free Glb and GNVs on this axis have not been previously investigated. A possible hypothesis is that increased amounts of miR-7-1-5p may contrast GLB-induced PPARγ overexpression. However, this can have implications both for the expression of NLRP3, that may be reduced by miR-7-1-5p, and miR-223-3p, as a decreased PPAR activity may reduce its concentration. Studies on other miRNAs targeting critical molecules involved in inflammasome activation will be needed to increase our understanding of this complex pathway. A limitation of our study is the lack of immunoblotting experiments, as well as evaluation of pro-IL-1β protein expression by specific monoclonal antibody.

Results herein confirm that free Glb inhibits the activation of the NLRP3 inflammasome, and shows a similar effect when loaded as GNVs, suggesting that GNV liposomes may be an efficient way to control activation of inflammasome and the release of proinflammatory cytokines. Because THP-1 cells are a well-known model to study the inflammatory response of monocytes/macrophages and the effect of anti-inflammatory compounds on inflammation [43], these findings allow the speculation that GNVs could have a similar anti-inflammatory effect in vivo. Validation in in vivo and ex vivo models and analysis of other miRNAs targeting critical molecules involved in inflammasome activation will be needed to increase our understanding of these complex pathways and to draw conclusions on a possible future therapeutic use of GNVs.

## 4. Materials and Methods

### 4.1. Cell Culture

Human acute monocytic leukemia THP-1 cells (IZSLER, Istituto Zooprofilattico Spermentale della Lombardia e dell’Emilia Romagna, Brescia, Italy) were maintained in culture medium (RPMI-1640) supplemented with L-glutamine, 10% heat-inactivated fetal bovine serum (FBS, PAN-Biotech, Aidenbach, Germany), 100 U/mL penicillin, and 100 μg/mL streptomycin (Invitrogen, Ltd., Paisley, UK). To favor differentiation into macrophages, cells plated in 24-well plates at 0.5 × 10^6^/well in culture medium containing PMA (200 nM) were incubated for 12 h in a humidifier cell incubator at 5% CO_2_ and 37 °C.

After removal of non-adherent cells by pre-warmed medium washing, THP-1 DMs were stimulated with LPS (1 µg/mL) and nigericine (50 µM) in the presence/absence of GNVs (10 µM) or Glb (25 µM). Negative (cells cultured in medium alone, or unstimulated) and positive (LPS+nigericine-stimulated cells) controls were used in every experiment. In preliminary experiments, gene expression of NLRP3 genes was analyzed at 1, 3, 6, and 24 h after activation; in subsequent experiments, gene and miRNA expression was measured at 1 h post activation, unless otherwise specified. Cell viability was verified by trypan blue exclusion [44] using a TC20 Automatic Cell Counter (Bio-Rad, Hercules, CA, USA).

### 4.2. Intracellular ASC, IL-1β, and NLRP3 Protein Staining

THP-1 DMs were permeabilized with 100 μL of saponin in PBS (0.1%) (Life Science VWR, Lutterworth, Leicestershire, UK). Then, 5 μL (25 μg/mL) of the PE-anti human ASC (clone HASC-71, isotype mouse IgG1, Biolegend, San Diego, CA, USA) or Alexa Fluor^®^ 488-anti human IL-b (clone 8516, isotype Mouse IgG1, R&D Systems, Minneapolis, MN, USA) or Alexa Fluor^®^ 488-anti human NLRP3 (Clone 768319, isotype RatIgG2a, R&D Systems, Minneapolis, MN, USA) monoclonal antibody was added to the tubes for 1 h at 4 °C. Cells were then washed with PBS and centrifuged at 1500× *g* for 10 min at 4 °C. Finally, cells were fixed with 100 μL of PFA in PBS (1%) (BDH, Walsall, UK) for 15 min, washed with PBS, centrifuged at 1500× *g* for 10 min at 4 °C, resuspended in 50 μL of iced PBS, and analyzed by AMNIS FlowSight (Luminex, Austin, TX, USA).

The following mAbs were used: PE-anti human ASC (clone HASC-71, isotype mouse IgG1, Biolegend, San Diego, CA, USA), Alexa Fluor^®^ 488-anti human IL-1β (clone 8516, isotype Mouse IgG1, R&D Systems), and Alexa Fluor^®^ 488-anti human NLRP3 (Clone 768319, isotype RatIgG2a, R&D Systems).

### 4.3. Image Stream Analysis by FlowSight AMNIS

THP-1 DMs resuspended in 50 µL of iced PBS were examined using the AMNIS FlowSight. Results were analyzed by IDEAS analysis software, version 6.0 (Amnis Corporation, Seattle, WA, USA). ASC-speck formation was analyzed by internalization, utilizing a mask that represents the whole cell defined by the bright field (BF) image and an internal mask defined by eroding the whole cell mask, and differentiating diffuse or spot (speck) fluorescence inside cells. A threshold mask was used to separate ASC-positive cells into ASC-speck spot cells and ASC-diffuse cells by the different diameter of the spot area: in ASC-speck, the spot shows a small area and high max pixel, and the opposite occurs in ASC-diffuse cells.

### 4.4. Flow Cytometry Analysis

Analyses were performed using a Beckman-Coulter (Brea, CA, USA) GALLIOS flow cytometer equipped with a 22 mW Blue Solid State Diode laser operating at 488 nm, and with a 25 mW Red Solid State Diode laser operating at 638 nm and interfaced with Kaluza analysis software. Flow cytometry compensation was performed using the fluorescence minus one (FMO) approach. Briefly, all antibody conjugates in the experiment are included except the one that is controlled for. FMO measures the spread of fluorescence from other staining parameters into the channel of interest, determining the threshold for positive staining.

### 4.5. Cytokine Production

Supernatants from THP-1 DMs activated for 24 h were collected, centrifuged, and stored at −20 °C. Caspase 1, IL-1β, and IL-18 concentrations were assessed in triplicate by Simple Plex assays on the ELLA microfluidic immunoassay system (ProteinSimple, San Jose, CA, USA). According to manufacturer’s instructions, 50 μL of diluted supernatants (1:1 ratio in sample diluent) was added to each sample inlet on the ELLA cartridge, in which microfluidic channels are located. Each channel contains three glass nanoreactors (GNRs) coated with a capture antibody; in this way, each sample was automatically processed in triplicate. Concentrations were generated from factory-calibrated standard curves pre-loaded into the cartridge. Sample results were reported using Simple Plex Runner v.3.7.2.0 (ProteinSimple). The limit of detection (LOD) was: caspase 1 0.04 pg/mL, IL-1β 0.064 pg/mL, and IL-18 0.2 pg/mL.

### 4.6. RNA Extraction

Total RNA (including miRNA and mRNA sequences) was extracted using the “miRNeasy cell and tissue Micro kit” (Qiagen, Hilden, Germany), according to the manufacturer’s instructions. Briefly, after homogenization in lysis buffer, total RNA extraction was performed using the semi-automated robot-work station Qiacube (Qiagen). Concentration was measured with a Qubit 2.0 fluorometer using the Qubit^®^ miRNA and RNA HS assays (High Sensitivity, ThermoFisher, Foster City, CA, USA).

### 4.7. miRNAs Quantitative Analysis by ddPCR

miRNAs were transcripted in cDNA with the miRCURY LNA RT Kit (Qiagen) in a total volume of 10 µL, according to the manufacturer’s protocol. miR-223-3p and miR-7-1-5p expression was analyzed by digital droplet PCR (ddPCR) using the ddPCR QX200 system (Bio-Rad). Briefly, 3 μL of diluted cDNA (1:500) and LNA^TM^-specific primers (Qiagen) were mixed with ddPCR EvaGreen Supermix (Bio-Rad); ddPCR workflow and data analyses were performed as previously reported [45]. A no template control and a negative control for each reverse transcription reaction were included in every assay to check for non-specific amplification. The number of copies/well evaluated using Quantasoft software analysis (Bio-Rad, version 1.7.4.0917) was converted in copies/ng of input RNA.

### 4.8. Gene Expression Quantitative Analysis by ddPCR

Inflammasome-related gene (NLRP3 and IL-1β) expression was analyzed by a one-step RT-ddPCR multiple assay with specific primers and fluorescent probes. In this study, 5 μL of extracted RNA (diluted 1:100) was added in the 20 μL of reaction mixtures containing 5 μL of 4× One-Step RT-ddPCR Supermix (Bio-Rad), 2 μL of reverse transcriptase (20 U/μL), 1 μL of 15 mM DTT, 1 μL for each target-specific assay (20×; final concentration: 250 nM probes, 900 nM for primers), and 5 μL of H_2_O. The workflow was similar to that described above, with generation of droplets and amplification (conditions: 60 min reverse transcription at 50 °C, 5 min at 95 °C, 45 cycles: 30 s at 95 °C and 55 s at 60 °C; 10 min at 98 °C) and reading of positive or negative droplets using the QX 200 droplet reader. Positive droplets were discriminated from negative droplets by a fluorescence amplitude threshold, set manually, using QuantaSoft software analysis (Bio-Rad version 1.7.4.0917). Samples were tested in triplicate; the reaction was considered positive if at least three droplets (out of 20,000 produced in the reaction) were positive. Number of copies/well was converted to copies/ng of input RNA.

### 4.9. Statistical Analysis

Experiments were repeated at least three times. Non-parametric expression data are reported as median and interquartile range (IQR: 25th and 75th percentiles). Statistical significance was calculated using the Kruskal–Wallis and Mann–Whitney U tests, or with the Wilcoxon signed-rank test for paired data. A level of *p* < 0.05 was considered statistically significant. Statistical analysis was performed using the MEDCALC software (version 14.10.2, Ostend, Belgium).

## 5. Conclusions

Overall, data presented herein show that GNVs, similarly to free Glb, down regulates NLRP3-mediated inflammation in an in vitro model and shed light on the possible effect of miR-7-1-5p and miR-223-3p in modulating this effect.

## Data Availability

Data is contained within the article and supplementary material.

## Supplementary Materials

The following supporting information can be downloaded at: https://www.mdpi.com/article/10.3390/ph16111590/s1. Figure S1: Representative dot plots and images of ASC-speck formation in THP-1 derived macrophages (DMs) in presence/absence of Glb or GNVs. Figure S2: Effect of GNVs on NLRP3 gene expression in THP-1 derived macrophages (DMs). NLRP3 mRNA expression during 24 h in LPS+nigericine (NIG)-activated THP-1 DMs in presence or absence of glibenclamide loaded nanovectors (GNVs) or free glibenclamide (Glb) in comparison to unstimulated cells. Table S1: NLRP3 gene expression in THP-1 DMs treated with different experimental conditions. Table S2: NLRP3-related miR-223-3p and miR-7-1-5p expression in THP-1 DMs treated with different experimental conditions.

## References

[1] Lamkanfi M., Mueller J.L., Vitari A.C., Misaghi S., Fedorova A., Deshayes K., Lee W.P., Hoffman H.M., Dixit V.M. (2009). Glyburide Inhibits the Cryopyrin/Nalp3 Inflammasome. J. Cell Biol..

[2] Simard J.M., Geng Z., Woo S.K., Ivanova S., Tosun C., Melnichenko L., Gerzanich V. (2009). Glibenclamide Reduces Inflammation, Vasogenic Edema, and Caspase-3 Activation after Subarachnoid Hemorrhage. J. Cereb. Blood Flow Metab..

[3] Lahmann C., Kramer H.B., Ashcroft F.M. (2015). Systemic Administration of Glibenclamide Fails to Achieve Therapeutic Levels in the Brain and Cerebrospinal Fluid of Rodents. PLoS ONE.

[4] Juhairiyah F., de Lange E.C.M. (2021). Understanding Drug Delivery to the Brain Using Liposome-Based Strategies: Studies That Provide Mechanistic Insights Are Essential. AAPS J..

[5] Poudel P., Park S. (2022). Recent Advances in the Treatment of Alzheimer’s Disease Using Nanoparticle-Based Drug Delivery Systems. Pharmaceutics.

[6] Giofrè S., Renda A., Sesana S., Formicola B., Vergani B., Leone B.E., Denti V., Paglia G., Groppuso S., Romeo V. (2022). Dual Functionalized Liposomes for Selective Delivery of Poorly Soluble Drugs to Inflamed Brain Regions. Pharmaceutics.

[7] Mikhailov M.V., Mikhailova E.A., Ashcroft S.J. (2001). Molecular Structure of the Glibenclamide Binding Site of the Beta-Cell K(ATP) Channel. FEBS Lett..

[8] Kimberly W.T., Bevers M.B., Von Kummer R., Demchuk A.M., Romero J.M., Elm J.J., Hinson H.E., Molyneaux B.J., Simard J.M., Sheth K.N. (2018). Effect of IV Glyburide on Adjudicated Edema Endpoints in the GAMES-RP Trial. Neurology.

[9] Sheth K.N., Petersen N.H., Cheung K., Elm J.J., Hinson H.E., Molyneaux B.J., Beslow L.A., Sze G.K., Simard J.M., Kimberly W.T. (2018). Long-Term Outcomes in Patients Aged ≤70 Years with Intravenous Glyburide from the Phase II GAMES-RP Study of Large Hemispheric Infarction: An Exploratory Analysis. Stroke.

[10] Kelley N., Jeltema D., Duan Y., He Y. (2019). The NLRP3 Inflammasome: An Overview of Mechanisms of Activation and Regulation. Int. J. Mol. Sci..

[11] Zhang G., Lin X., Zhang S., Xiu H., Pan C., Cui W. (2017). A Protective Role of Glibenclamide in Inflammation-Associated Injury. Mediators Inflamm..

[12] Bauernfeind F., Rieger A., Schildberg F.A., Knolle P.A., Schmid-Burgk J.L., Hornung V. (2012). NLRP3 Inflammasome Activity Is Negatively Controlled by miR-223. J. Immunol..

[13] Zhou Y., Lu M., Du R.-H., Qiao C., Jiang C.-Y., Zhang K.-Z., Ding J.-H., Hu G. (2016). MicroRNA-7 Targets Nod-like Receptor Protein 3 Inflammasome to Modulate Neuroinflammation in the Pathogenesis of Parkinson’s Disease. Mol. Neurodegener..

[14] Kim J.K., Jin H.S., Suh H.-W., Jo E.-K. (2017). Negative Regulators and Their Mechanisms in NLRP3 Inflammasome Activation and Signaling. Immunol. Cell Biol..

[15] Kawahara Y., Kaneko T., Yoshinaga Y., Arita Y., Nakamura K., Koga C., Yoshimura A., Sakagami R. (2020). Effects of Sulfonylureas on Periodontopathic Bacteria-Induced Inflammation. J. Dent. Res..

[16] Saresella M., La Rosa F., Piancone F., Zoppis M., Marventano I., Calabrese E., Rainone V., Nemni R., Mancuso R., Clerici M. (2016). The NLRP3 and NLRP1 Inflammasomes Are Activated in Alzheimer’s Disease. Mol. Neurodegener..

[17] Piancone F., Saresella M., Marventano I., La Rosa F., Santangelo M.A., Caputo D., Mendozzi L., Rovaris M., Clerici M. (2018). Monosodium Urate Crystals Activate the Inflammasome in Primary Progressive Multiple Sclerosis. Front. Immunol..

[18] Piancone F., Saresella M., La Rosa F., Marventano I., Meloni M., Navarro J., Clerici M. (2021). Inflammatory Responses to Monomeric and Aggregated α-Synuclein in Peripheral Blood of Parkinson Disease Patients. Front. Neurosci..

[19] Blevins H.M., Xu Y., Biby S., Zhang S. (2022). The NLRP3 Inflammasome Pathway: A Review of Mechanisms and Inhibitors for the Treatment of Inflammatory Diseases. Front. Aging Neurosci..

[20] La Rosa F., Saresella M., Marventano I., Piancone F., Ripamonti E., Al-Daghri N., Bazzini C., Zoia C.P., Conti E., Ferrarese C. (2019). Stavudine Reduces NLRP3 Inflammasome Activation and Modulates Amyloid-β Autophagy. J. Alzheimers Dis..

[21] La Rosa F., Mancuso R., Agostini S., Piancone F., Marventano I., Saresella M., Hernis A., Fenoglio C., Galimberti D., Scarpini E. (2021). Pharmacological and Epigenetic Regulators of NLRP3 Inflammasome Activation in Alzheimer’s Disease. Pharmaceuticals.

[22] La Rosa F., Zoia C.P., Bazzini C., Bolognini A., Saresella M., Conti E., Ferrarese C., Piancone F., Marventano I., Galimberti D. (2022). Modulation of MAPK- and PI3/AKT-Dependent Autophagy Signaling by Stavudine (D4T) in PBMC of Alzheimer’s Disease Patients. Cells.

[23] Li C., Levin M., Kaplan D.L. (2016). Bioelectric Modulation of Macrophage Polarization. Sci. Rep..

[24] Pétrilli V., Papin S., Dostert C., Mayor A., Martinon F., Tschopp J. (2007). Activation of the NALP3 Inflammasome Is Triggered by Low Intracellular Potassium Concentration. Cell Death Differ..

[25] Xu Z., Liu Y., Yang D., Yuan F., Ding J., Wang L., Qu M., Yang G., Tian H. (2017). Glibenclamide–Sulfonylurea Receptor 1 Antagonist Alleviates LPS-Induced BV2 Cell Activation through the P38/MAPK Pathway. RSC Adv..

[26] Kim J., Park J.-H., Shah K., Mitchell S.J., Cho K., Hoe H.-S. (2021). The Anti-Diabetic Drug Gliquidone Modulates Lipopolysaccharide-Mediated Microglial Neuroinflammatory Responses by Inhibiting the NLRP3 Inflammasome. Front. Aging Neurosci..

[27] Yang J., Yang J., Huang X., Xiu H., Bai S., Li J., Cai Z., Chen Z., Zhang S., Zhang G. (2022). Glibenclamide Alleviates LPS-Induced Acute Lung Injury through NLRP3 Inflammasome Signaling Pathway. Mediators Inflamm..

[28] Kominato Y., Galson D., Waterman W.R., Webb A.C., Auron P.E. (1995). Monocyte Expression of the Human Prointerleukin 1 Beta Gene (IL1B) Is Dependent on Promoter Sequences Which Bind the Hematopoietic Transcription Factor Spi-1/PU.1. Mol. Cell Biol..

[29] Adamik J., Wang K.Z.Q., Unlu S., Su A.-J.A., Tannahill G.M., Galson D.L., O’Neill L.A., Auron P.E. (2013). Distinct Mechanisms for Induction and Tolerance Regulate the Immediate Early Genes Encoding Interleukin 1β and Tumor Necrosis Factor α. PLoS ONE.

[30] Ma T., Liu X., Cen Z., Xin C., Guo M., Zou C., Song W., Xie R., Wang K., Zhou H. (2018). MicroRNA-302b Negatively Regulates IL-1β Production in Response to MSU Crystals by Targeting IRAK4 and EphA2. Arthritis Res. Ther..

[31] Tapia V.S., Daniels M.J.D., Palazón-Riquelme P., Dewhurst M., Luheshi N.M., Rivers-Auty J., Green J., Redondo-Castro E., Kaldis P., Lopez-Castejon G. (2019). The Three Cytokines IL-1β, IL-18, and IL-1α Share Related but Distinct Secretory Routes. J. Biol. Chem..

[32] Zamani P., Oskuee R.K., Atkin S.L., Navashenaq J.G., Sahebkar A. (2020). MicroRNAs as Important Regulators of the NLRP3 Inflammasome. Prog. Biophys. Mol. Biol..

[33] Yuan X., Berg N., Lee J.W., Le T.-T., Neudecker V., Jing N., Eltzschig H. (2018). MicroRNA miR-223 as Regulator of Innate Immunity. J. Leukoc. Biol..

[34] Chen Q., Wang H., Liu Y., Song Y., Lai L., Han Q., Cao X., Wang Q. (2012). Inducible microRNA-223 down-Regulation Promotes TLR-Triggered IL-6 and IL-1β Production in Macrophages by Targeting STAT3. PLoS ONE.

[35] Zhao J., Zhou Y., Guo M., Yue D., Chen C., Liang G., Xu L. (2020). MicroRNA-7: Expression and Function in Brain Physiological and Pathological Processes. Cell Biosci..

[36] Bravo-Egana V., Rosero S., Molano R.D., Pileggi A., Ricordi C., Domínguez-Bendala J., Pastori R.L. (2008). Quantitative Differential Expression Analysis Reveals miR-7 as Major Islet microRNA. Biochem. Biophys. Res. Commun..

[37] Correa-Medina M., Bravo-Egana V., Rosero S., Ricordi C., Edlund H., Diez J., Pastori R.L. (2009). MicroRNA miR-7 Is Preferentially Expressed in Endocrine Cells of the Developing and Adult Human Pancreas. Gene Expr. Patterns.

[38] Chen H., Guo M., Yue D., Zhao J., Zhou Y., Chen C., Liang G., Xu L. (2021). MicroRNA-7 Negatively Regulates Toll-like Receptor 4 Signaling Pathway through FAM177A. Immunology.

[39] Li C.H., Gong D., Chen L.Y., Zhang M., Xia X.D., Cheng H.P., Huang C., Zhao Z.W., Zheng X.L., Tang X.E. (2017). Puerarin promotes ABCA1-mediated cholesterol efflux and decreases cellular lipid accumulation in THP-1 macrophages. Eur. J. Pharmacol..

[40] Odegaard J.I., Ricardo-Gonzalez R.R., Goforth M.H., Morel C.R., Subramanian V., Mukundan L., Red Eagle A., Vats D., Brombacher F., Ferrante A.W. (2007). Macrophage-Specific PPARgamma Controls Alternative Activation and Improves Insulin Resistance. Nature.

[41] Bouhlel M.A., Derudas B., Rigamonti E., Dièvart R., Brozek J., Haulon S., Zawadzki C., Jude B., Torpier G., Marx N. (2007). PPARgamma Activation Primes Human Monocytes into Alternative M2 Macrophages with Anti-Inflammatory Properties. Cell Metab..

[42] Ying W., Tseng A., Chang R.C.-A., Morin A., Brehm T., Triff K., Nair V., Zhuang G., Song H., Kanameni S. (2015). MicroRNA-223 Is a Crucial Mediator of PPARγ-Regulated Alternative Macrophage Activation. J. Clin. Investig..

[43] Chanput W., Mes J.J., Wichers H.J. (2014). THP-1 Cell Line: An in Vitro Cell Model for Immune Modulation Approach. Int. Immunopharmacol..

[44] Mosmann T. (1983). Rapid Colorimetric Assay for Cellular Growth and Survival: Application to Proliferation and Cytotoxicity Assays. J. Immunol. Methods.

[45] Mancuso R., Agostini S., Hernis A., Caputo D., Galimberti D., Scarpini E., Clerici M. (2022). Alterations of the miR-126-3p/POU2AF1/Spi-B Axis and JCPyV Reactivation in Multiple Sclerosis Patients Receiving Natalizumab. Front. Neurol..

