# Supplementary material for: Glibenclamide-Loaded Nanoparticles Reduce NLRP3 Inflammasome Activation and Modulate miR-223-3p/miR-7-1-5p Expression in THP-1 Cells
Source: Pharmaceuticals (Basel). 2023 Nov 10;16(11):1590. doi: 10.3390/ph16111590 (PMC10675475; doi:10.3390/ph16111590)
Supplement: Supplementary file 1 [file pharmaceuticals-16-01590-s001.zip › pharmaceuticals-2699183-supplementary.pdf]

Glibenclamide-loaded nanoparticles reduce NLRP3 inflammasome activation and modulate miR-223-3p/miR-7-1-5p expression in THP-1 cells

Roberta Mancuso[1], Lorenzo Agostino Citterio[1], Simone Agostini[1], Ivana Marventano[1], Francesca La Rosa[1], Francesca Re[3], Pierfausto Seneci[4], Marina Saresella[1], Mario Clerici[1,2]

[1]IRCCS Fondazione Don Gnocchi – ONLUS, 20148, Milan, Italy
[2]Department of Pathophysiology and Transplantation, University of Milan, 20122, Milan, Italy
[3]School of Medicine and Surgery, University of Milano-Bicocca, 20854 Vedano al Lambro, Italy
[4]Dipartimento di Chimica, University of Milan, 20133 Milan, Italy

Supplementary Materials

Table S1. NLRP3 and pro-IL-1β gene expression in THP-1 DM treated with different experimental conditions. For each gene, mRNA concentration was measured by ddPCR after 1 hour of incubation with: LPS+ nigericine, glibenclamide loaded nanovector (GNV) or glibenclamide (Glb). Data are expressed as copies/ng of extracted RNA. Median and IQR (25th and 75th percentiles) are showed.

	**NLRP3 (copies/ng)**	**pro-IL-1β (copies/ng)**
**UNSTIMULATED**	578.36 (463.69-591.95)	362.12 (343.55-399.87)
**LPS+NIGERICINE**	3874.36 (3845.62-3935.73)	1685.23 (1664.32-1700.42)
**Glb**	408.79 (405.36-422.58)	1165.32 (1126.65-1220.43)
**Glb+LPS +NIGERICINE**	400.12 (389.25-408.58)	923.91 (921.38-925.64)
**GNV**	487.63 (421.93-561.14)	348.76 (342.01-359.61)
**GNV+LPS+NIGERICINE**	987.36 (930.00-1130.00)	5000.00 (4908.91-5025.94)

Table S2. NLRP3 related miR-223-3p and miR-7-1-5p expression in THP-1 DM treated with different experimental conditions. miRNA concentration was measured by ddPCR after 1 hour of incubation with: LPS + nigericine, glibenclamide loaded nanovector (GNV), glibenclamide (Glb). Data are expressed as copies/ng of extracted RNA and as median and IQR (25th and 75th percentiles).

	**miR-223-3p (copies/ng)**	**miR-7-1-5p (copies/ng)**
**UNSTIMULATED**	346,523.25 (300117.12–359379.56)	750,12 (739.66-759.11)
**LPS+NIGERICINE**	71,165.36 (71151.80-71187.44)	12,870.36 (12,573.77-12,988.78)
**Glb**	860.81 (857.04-874.83)	606,125 (605,516.78-606,242.16)
**Glb+LPS +NIGERICINE**	3185.36 (3106.58-3199-93)	424,065.36 (422,444.07-424,225.20)
**GNV**	299,800.60 (295,933.33-318,761.00)	725,015.32 (724,966.07-730,638.83)
**GNV+LPS+NIGERICINE**	1098.25 (1009.83-1106.80)	1,276,984.34 (1,272,684.34-1,287,199.66)

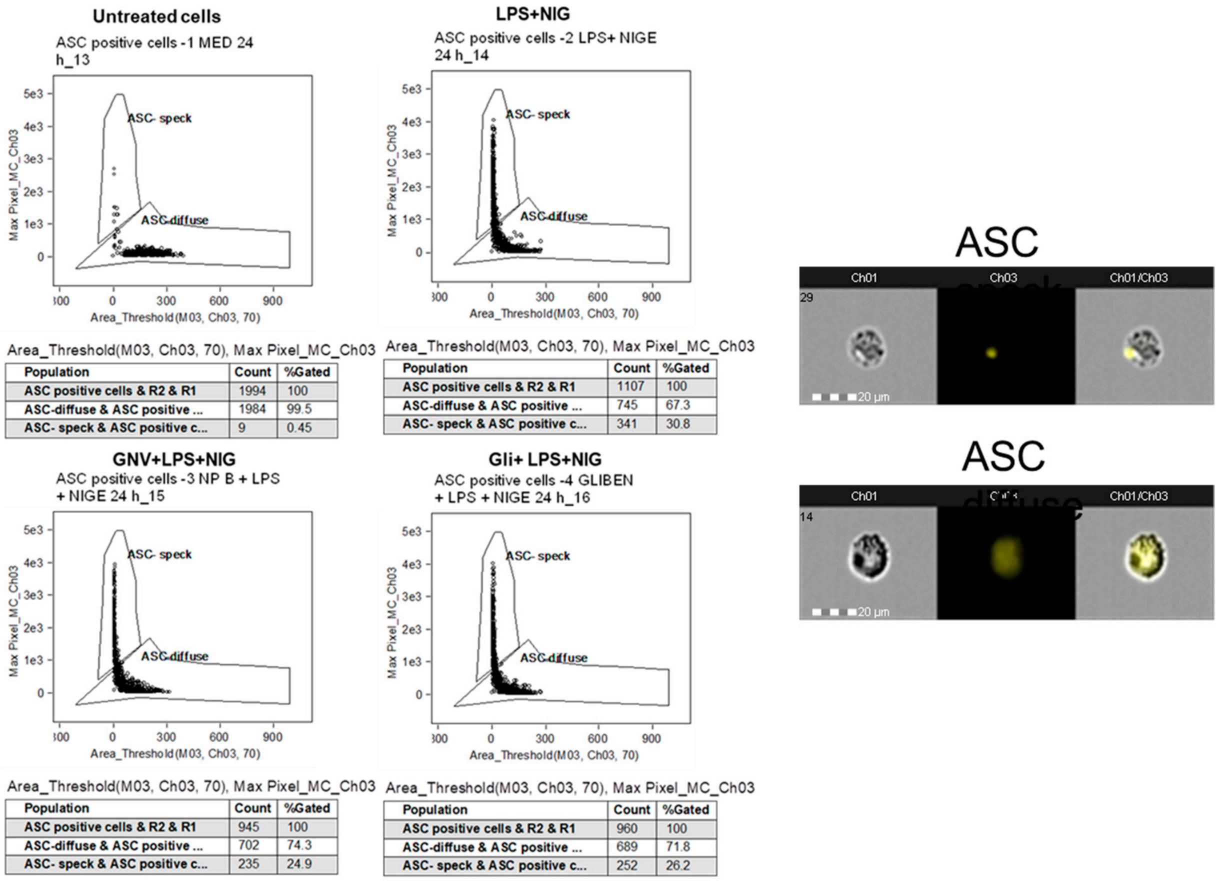

Figure S1. (a) Representative dot plots of THP-1 DM expressing ASC-speck formation in unstimulated or LPS+NIG stimulated cells in presence/absence of Glb or GNV. ASC positive cell are plotted on Max Pixel MC (Ch03) *vs.* area threshold (M03, Ch03,70) scatter plot. This mask allows for discriminating between cells in which ASC-specks are formed and a functional inflammasome complex is assembled, and those with an ASC diffuse pattern. (**b**) Representative gallery images show ASC-speck (upper) and ASC diffuse (bottom) THP-1 DM. The first column shows cells in brightfield (BF), the second column shows ASC-PE fluorescence, the third column shows florescence of ASC merged with BF (IDEA software).

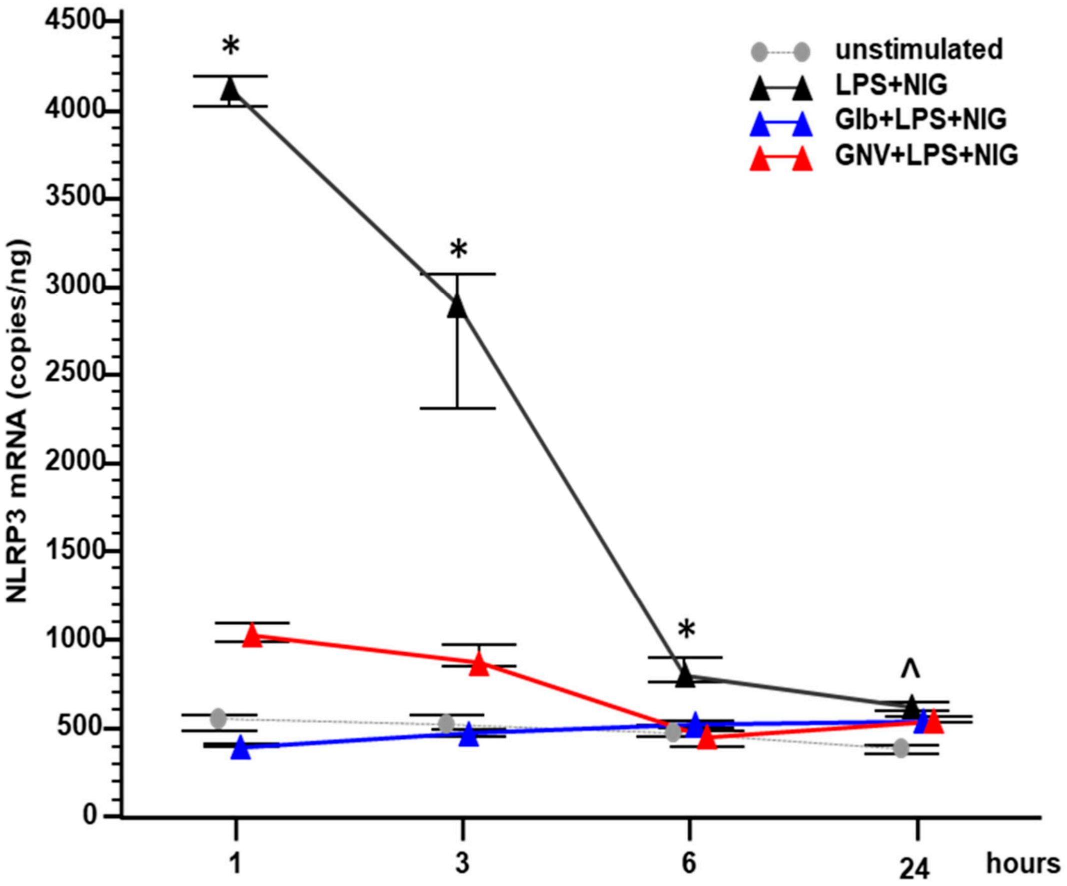

Figure S2. Effect of GNV on NLRP3 mRNA expression in THP-1 DM cells. Results obtained in LPS+nigericine (NIG) activated THP-1 DM cells in presence/absence of glibenclamide loaded nanovector (GNV) or glibenclamide (Glb) are shown. Unstimulated: THP-1 DM cells cultured alone. Data are expressed as copies/ng. Median values and interquartile range (25th - 75th percentiles) of three independent experiments were shown. *= $p<0.01$ for all the conditions *vs.* LPS+NIG. ^= $p<0.01$ for untreated *vs.* LPS+NIG only.
